# Culture and emotion perception: comparing Canadian and Japanese children’s and parents’ context sensitivity

**DOI:** 10.1007/s40167-017-0052-0

**Published:** 2017-07-28

**Authors:** Hajin Lee, Kristina Nand, Yuki Shimizu, Akira Takada, Miki Kodama, Takahiko Masuda

**Affiliations:** 1 Department of Psychology, University of Alberta, Edmonton, AB T6G 2E9 Canada; 2 University of Tokyo, Tokyo, Japan; 3 Saitama University, Saitama, Japan; 4 Kyoto University, Kyoto, Japan

**Keywords:** Culture, Attention, Emotion, Reasoning, Canadians, Japanese, Parental guidance

## Abstract

Prior research on the perception of facial expressions suggests that East Asians are more likely than North Americans to incorporate the expressions of background figures into their judgment of a central figure’s emotion (Masuda et al. in J Pers Soc Psychol 94:365–381, 2008b). However, little research has examined this issue in the context of developmental science, especially during joint sessions where parents engage in a task in front of their 7–8-year-old children. In this study, 22 Canadian and 20 Japanese child-parent dyads participated in an emotion judgment task, and were asked to judge a central figure’s emotion and explain their reasoning. The results indicated that while early elementary school children did not show culturally dominant reasoning styles, parents displayed culturally dominant modes of attention, serving as models for their children.

## Introduction

A plethora of evidence in the literature of cultural psychology has suggested that there are systematic cultural variations in cognition and perception across young adult members: North Americans dominantly display an analytic and object-oriented mode of attention, whereas East Asians, such as Chinese, Japanese, and Koreans, dominantly display a holistic and context-oriented mode of attention, and these culturally dominant modes have developed through historically evolved worldviews and epistemologies (e.g., Masuda et al. 2017c; Nisbett 2003; Nisbett and Masuda 2003; Nisbett and Miyamoto 2005; Nisbett et al. 2001).

Accumulating empirical studies have given credence to these suggestions, demonstrating that such cultural variations in modes of attention are observable when using abstract shape stimuli such as the framed line task (Duffy et al. 2009; Ji et al. 2000; Kitayama et al. 2003; Masuda et al. 2008a; Uskul et al. 2008), concrete object images such as wildlife, fish, and vehicles (Chua et al. 2005; Imada et al. 2013; Masuda and Nisbett 2001, 2006; Senzaki et al. 2014, 2016), and visual representations such as drawings, paintings, collages, and photographs (Masuda et al. 2008c; Nand et al. 2014; Ueda and Komiya 2012; Wang et al. 2012). Although cultural differences in modes of attention have been shown to be robust among young adults, cultural psychologists have only recently explored this issue in child development, in particular when and how children learn such culturally unique attentional styles (Masuda et al. 2017b; Senzaki et al. 2014, 2016). Assuming that interactions between parents and children may play a key role in understanding the influence of culture on child development, the present study aims to examine both children’s and parents’ modes of attention, i.e., their perception of facial expressions embedded in a social context.

### Culture and emotional perception in social contexts

The effect of context sensitivity on the perception of facial expressions has been debated in the field of emotion research. On one hand, several studies have indicated that in certain contexts which contain salient information, such as a threat, North Americans showed heightened attention to the contexts surrounding them (Baumann and DeSteno 2010; Carroll and Russell 1996; Carroll and Young 2005; Fazio 2001; Hietanen et al. 2007; Leppänen and Hietanen 2003; Olson and Marshuetz 2005; Russell 1991; Russell and Fehr 1987; Strick et al. 2009), and sometimes their degree of context sensitivity became comparable to that of East Asians when they were exposed to affective priming (Ito et al. 2012).

Nevertheless, cross-cultural findings in this domain have provided evidence that the degree of context sensitivity is in general stronger for East Asians than for North Americans in an emotion judgment task (e.g., Ito et al. 2013; Masuda et al. 2008b, 2012). For example, Masuda et al. (2008b) asked European American and Japanese undergraduate students to observe several cartoon images of a center figure with two background figures standing on each side. The four background figures had either congruent or incongruent facial expressions to the central figure. Participants were then asked to judge the center figure’s emotion based on his/her facial expression. The results showed that East Asians considered the background figures’ facial expression more than North Americans by incorporating the background information into their judgment of the target figure’s emotion.

In particular, when presented with congruent emotional images where both the center and the background figures had the same facial expressions (either happy or sad faces), East Asians, as holistic and context-oriented thinkers, tended to perceive the center figures emotion (happiness or sadness) as intensified compared to incongruent emotional images where the center figure showed different facial expressions from the surrounding figures’ facial expressions (e.g., happy face for the center figure and sad faces for the background faces). In contrast, North Americans, as analytic and object-oriented thinkers, exclusively focused on the center figure’s facial expressions and were unaffected by the background figures’ facial expressions.

### Child development and modes of attention

Understanding when cultural differences in context sensitivity emerge in child development is crucial for understanding cognitive development, as well as socialization processes, in a given culture (e.g., Maccoby 1984). Researchers have recently explored developmental trajectories of culturally divergent patterns of attention among preschoolers and early elementary children. These findings in developmental science converge with the findings in cultural psychology, suggesting that culturally divergent modes of attention emerge gradually over time, with some variability depending on the difficulty of the task. When the tasks did not involve children’s advanced linguistic and reasoning skills (e.g., drawing pictures, coloring pictures, and simply labeling facial expressions), cultural variations in attention emerged before age 6 (e.g., Ishii et al. 2014; Kuwabara and Smith 2012, 2016; Kuwabara et al. 2011; Richland et al. 2010). However, when the tasks involved children’s advanced linguistic and reasoning skills (e.g., describing pictorial images or movies and explaining the reasons for their judgments), cultural differences became salient at around age 10 (e.g., Ji 2008; Masuda et al. 2017b; Miller 1984; Senzaki et al. 2016). For example, Masuda et al. (2017b) investigated the emergence of culturally unique modes of attention in children’s developmental trajectories by using the emotion judgment task (Masuda et al. 2008b) and confirmed that Japanese children at age 10 showed greater context sensitivity compared to their Canadian counterparts. Notably, their emotion judgment corresponded with the reasoning for their judgment.

In addition to the investigation of developmental trajectories, in which cultural variation in modes of attention emerge, another line of research explores how children learn such culturally dominant modes of attention through interaction with their parents: how parents transmit cultural skills or knowledge to their children, and how children internalize such skills and knowledge. Some theoretical discourses explain human nature from an evolutionary perspective, proposing that humans accumulate cultural knowledge by transmitting it from one generation to the next, by sharing it, modifying it, and adding new knowledge to the accumulated cultural knowledge (e.g., Mesoudi 2011; Richerson and Boyd 2005; Tomasello 1999, 2009). Other theoretical discourses argue for the influence of a socio-cultural environment on an individual’s cultural learning through interactions with others (e.g., Cole 1998; Duranti et al. 2011; Gauvain 2001; Rogoff 2003; Vygotsky 1978; Wood et al. 1976). We hold the cultural co-evolution perspective, which affirms both discourses and highlights their interconnections. Together, they address the importance of the parental role in conveying culturally important meaning systems, practices, habits, and associated psychological processes for children to survive in a given cultural milieu.

Based on these theoretical discourses, empirical studies in developmental science have investigated how culture shapes parental behavior and how children learn culturally dominant skills and knowledge through interaction with their parents (e.g., Bornstein et al. 1992; Caudill and Weinstein 1969; Fernald and Morikawa 1993; Ishii et al. 2014; Senzaki et al. 2016; Wang 2001; Wang et al. 2000).

For example, Senzaki et al. (2016) found that when comparing two age groups of children (aged 4–6 and 7–9) and parents in Canada and Japan, parents assisted their children to attend to a task in a culturally unique way, using either object-oriented or context-sensitive attention. When children engaged in a visual description task alone, they did not show clear cultural modes of attention: both cultural groups showed object-oriented attention by mainly describing focal objects in the scene. However, when parents joined the task with their children, the children in the older-age group tended to demonstrate cultural modes of attention similar to their parents. That is, with parental support, Canadian children aged 7–9 consistently showed object-oriented attention, whereas Japanese children aged 7–9 described focal objects as well as contexts. However, those in the younger age group did not show such a tendency. Thus, this parent–child interaction could be effective only when children reach a certain level of cognitive and language ability, fostering children’s emerging cultural modes of attention. These studies converge to suggest that during parent–child interaction, parents transmit culturally important skills and knowledge to their children, such as how to attend to and explain social events.

### Overview of the present study

Extending from the aforementioned studies, we aimed to elucidate the relationships among culture, child development, and perception of facial expressions. However, to date, little research has discussed this issue in terms of whether parents indeed show culturally dominant attention styles when interacting with their children in an emotion judgment task (Masuda et al. 2017b). To answer this question, we conducted an experiment to examine both children’s behaviors on their own, and parents’ behaviors in front of their children.

We recruited 42 parent and child dyads at age 7–8 (*N* = 84) in Canada and in Japan. Consistent with previous findings by Masuda et al. (2017b) and Senzaki et al. (2016), we expected that 7–8-year-old children in both cultures would not show clear cultural variations in their attention styles. Parents, however, would still display such culturally dominant modes of attention when engaging in the task with their 7–8-year-old children as a parental guide. More specifically, we expected that Japanese parents would be more likely than Canadian parents to take into account the background figures’ facial expressions when judging the center figure’s emotion by referring to the background figures more frequently.

## Methods

### Participants

Twenty-two Canadian children (14 girls and 8 boys, *mean age* = 7.68, *SD* = .48) and 20 Japanese children (12 girls and 8 boys, *mean age* = 7.70, *SD* = .47) and their parents participated in the study. Parents in both cultures reported their child’s demographic information in a questionnaire. There was no significant difference in children’s mean age across two cultures, *F* < 1, *ns*. Canadian data was collected either in a small room at a sports or daycare facility, or a laboratory at the University of Alberta. All Canadian participants spoke English as their first language. One child participant was born in Vietnam, and the rest were born in Canada; however, all child participants were raised in Canada. Sixteen children were identified as European Canadians/Caucasian, one was identified as Arabic Canadian and Caucasian, one as European Canadian and Latina, one as First Nations and Caucasian, and one as Asian/Pacific Islander. Two children’s ethnic identity was unspecified. After the session, they received a $25 gift card for a toy store, a bookstore, or a sports shop as an honorarium for their participation. A comparable sample of Japanese data was collected at a laboratory at Saitama University and a laboratory at Kyoto University. All Japanese participants were born and raised in Japan, and spoke Japanese as their first language. They each received a 1000 Japanese Yen ($9) gift card for bookstores as the honorarium for their participation. All Canadian and Japanese children were accompanied by their parents. The parents from both cultures joined in the last half of the session after their children finished the initial session alone. Parents’ age, religion, and income information were not collected, but we asked parents to report their highest level of education completed. There was no difference in parental education level across cultures (mother’s education, *F* (1, 39) = 2.39, *p* = .13; father’s education, *F* < 1, *ns*).

### Materials

We selected images from Masuda et al. (2008b). Each image consisted of cartoon children—specifically, a center figure and four background figures. In total, 30 images consisting of 2 (center figures: Asian and Caucasian) × 5 (the center figure’s facial expressions: Strongly Happy, Moderately Happy, Strongly Sad, Moderately Sad, and Neutral) × 3 (the background figures’ facial expressions: Happy, Sad, and Neutral) cartoon children were used for the study. Following Masuda et al.’s (2008b) procedure, the 30 images were presented in two different orders (1–30 or 30–1). All the presentation timings of the images were administered using Microsoft PowerPoint (Microsoft, Co.). The entire session was video-recorded from the back of participants for later coding and to maintain their anonymity. The purpose of video recording was to transcribe participants’ accounts later and to confirm the stimulus presentation (i.e., which image the participants were looking at). Based on the transcriptions from these videos, we could code the data for both the foreground and background accounts.

In the experimental session, children and parents respectively observed each image for 5 s and were subsequently asked to judge the center figure’s level of happiness and sadness based on a 10-point Likert scale (Masuda et al. 2008b, 2017a, b). To facilitate young children’s understanding of the scale, we attached schematic images of faces underneath the scale, which were originally created by Ishii et al. (2016). When presented each image, participants selected a number from the scale. The experimenter then asked them to explain their reasoning regarding why they selected that number. To avoid the experimenter’s obtrusive influence on participants, we focused only on their initial responses to the question, and did not provide any additional comments or conversation.

### Procedure

Children independently engaged in the first 15 trials, and then engaged in the remaining 15 trials with their parents. Parents filled in the demographic questionnaire while their children engaged in the first 15 trials. After the children completed the initial trials, they were asked to help the experimenter by taking the experimenter’s role and asking their parent, who would now be in their place as the new participant, to complete the latter half of the trials by engaging in the same task involving judging the center figure’s level of happiness and sadness based on the Likert scale and explaining their answer. In order to control children’s influence on parents’ performance, we instructed children to simply ask the provided questions and not to engage in conversation with their parents. This procedure allowed us to examine whether parents showed culturally unique patterns of attention in front of their children. While the parent completed the remaining 15 trials alongside their child, the experimenter remained in the room but behind the scene in order to remain unobtrusive but available if either the parent or child had any questions or difficulty with the task.

We mainly focused on measuring the children’s and parents’ reasoning styles. Each time the child judged the intensity of the target person’s emotion, they were asked to explain their reasoning. Parents gave more accounts than children, and there were no cross-cultural differences between children or between parents in the number of accounts. The average length of the responses for children was around 20 words and the average length of the responses for parents was around 30 words across cultures. Canadian data was transcribed by native English speakers and Japanese data by native Japanese speakers. Adopting a coding strategy from previous research (Masuda et al. 2017b), each account was coded into two categories: (1) foreground accounts, including information regarding the target person’s emotion, facial expressions, and references to parts of the face; and (2) background accounts, including the background figures’ emotion, facial expressions and references to parts of the face, and relational explanations between the center figure and the background figures. Total scores for foreground and background accounts ranged from 0 to 30, with a score of 0–1 for each judgement (happiness or sadness) made during the 15 trials.

For instance, if a child simply stated, “He looks happy,” we would mark a value of 1 in the cell for a foreground account, but leave the cell for a background account blank. Statements that referred to the background figures, such as *his friends* and *the people in the back* in English (*tomodachi* and *mawarinohito* in Japanese) were categorized as a background account. Coders put a value of 1 in the cells for both foreground and background accounts when a child referred specifically to the central figure as well as the surrounding figures, such as, *all* and *everyone* in English (*minna* and *zen*-*in* in Japanese) or contrasted the central figure with the background, such as *only the center person* in English (*mannaka no hitodakeha* in Japanese).

One of the investigators coded the Canadian participants’ reasoning data, and another investigator coded the Japanese participants’ reasoning data. In addition, an English–Japanese bilingual coder who was blind to the experimental hypotheses coded all Canadian and Japanese data. The results of the reliability check indicated that intercoder agreement was 99.46% for Canadian data and 98.96% for Japanese data. Disagreements were resolved by discussion between coders.

### Results

We counterbalanced the stimulus presentation order: about half of the children began their trials with image 1, and the rest of children began with image 30. Similarly, about half of the parents started from image 1, and the rest of parents started with image 30. As the judgment data (the extent to which participants were influenced by changes in the background) was originally designed to be analyzed as a whole, it would have been more thorough to have both parents and children engage in 30 trials each. However, due to the time constraint, both parents and children completed only half of the trials (i.e., 15 trials). Identical trials were presented to both parents and children, consisting of the same center figures (either Asian or Caucasian) with emotional expressions of consistent intensity (i.e., three strongly happy expressions, three moderately happy expressions, three strongly sad expressions, three moderately sad expressions, and three neutral expressions). The only difference in stimuli between parents and children was that the ethnicity of the center figure alternated such that when a parent viewed an Asian center figure, their child viewed a Caucasian center figure with the same emotional expression of the same intensity and vice versa.

**Fig. 1:**
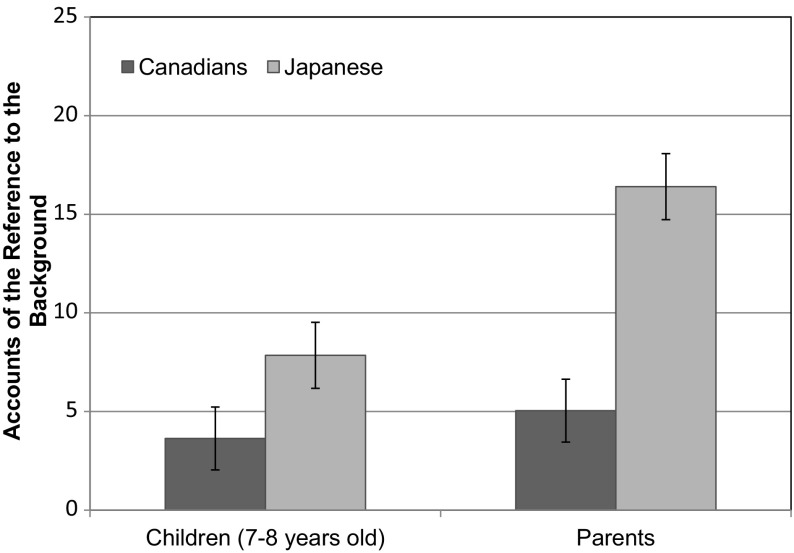
Children’s and parents’ accounts of the reference to the background. *Error bars* represent standard errors

Our design consisted of both Asian and Caucasian faces in order to investigate whether there was any difference in facial recognition of the center figure between different races. In fact, previous studies (Ito et al. 2012, 2013; Masuda et al. 2008b, 2012) and the present study found that there was no difference in facial recognition. The five labels of the center figure’s emotional expressions were used for our organization of the stimuli. Thus, they were irrelevant to the participants’ ratings of emotion perception. According to Masuda et al. (2008b), despite the fact that participants were exposed to a variety of emotional expressions, they may not have noticed the systematic structures of the combinations of the slightly different five expressions used for the center figure’s facial expressions and background figures’ expressions, which had only one expression per emotion.

In this task, children and parents only completed half the amount of trials required (when compared to the original), which is why the data is insufficient when it comes to analyzing the judgment scores. In addition, the order of stimulus presentation did not allow us to analyze the emotion judgment data. We counterbalanced the stimulus order in order to remove the order effect, and alleviate the above insufficiency. However, another paper confirmed that, consistent with previous findings, participants’ emotion judgment in general corresponded with the reasoning for their judgment (Masuda et al. 2017b). Therefore, the current study focused on only participants’ reasoning data as an indicator of one’s context sensitivity (i.e., their explanations of the foreground and background accounts for their judgment). Since there were no main effects of gender and presentation order, and no interaction effect between them, we collapsed these variables for the following analyses.

#### Children’s and parents’ accounts of the foreground reference

A 2 (Culture: Canada vs. Japan) × 2 (Group: Children vs. Parents) ANOVA was applied to the number of foreground accounts. The results showed that there was no main effects of culture, *F*(1, 80) = 1.82, *p* > .10, η_p_^2^ = .02, and group, *F*(1, 80) = 1.41, *p* > .20, η_p_^2^ = .02. The effect of the culture and group interaction was also not statistically significant, *F* < 1, *ns*. Because both Canadian and Japanese children and parents referred to the center figure to an equivalent extent, no significant differences were observed (Children: *M*_*CAN*_ = 29.59, *SD*_*CAN*_ = 1.71 vs. *M*_*JPN*_ = 29.05, *SD*_*JPN*_ = 1.10; Parents: *M*_*CAN*_ = 29.68, *SD*_*CAN*_ = .72 vs. *M*_*JPN*_ = 29.55, *SD*_*JPN*_ = .69). These results suggested that parents and their children in both cultures equally understood the task (i.e., judging the center figure’s emotion based on their facial expressions) and equally thought that the center figure’s facial expressions were important indicators to assess one’s emotion.

#### Children’s and parents’ accounts of the background reference

A 2 (Culture: Canada vs. Japan) × 2 (Group: Children vs. Parents) ANOVA was applied to the number of background references. The results indicated that there were main effects of culture, *F*(1, 80) = 22.69, *p* < .001, η_p_^2^ = .221, and group, *F*(1, 80) = 9.29, *p* = .003, η_p_^2^ = .104. However, these main effects were qualified by the interaction between culture and group, *F*(1, 80) = 4.78, *p* = .032, η_p_^2^ = .056. The simple effect analyses using multiple *t* tests without the assumption of variance equality revealed that while there were no cultural differences in the number of children’s accounts for the background reference (*M*_*CAN*_ = 3.64, *SD*_*CAN*_ = 6.34 vs. *M*_*JPN*_ = 7.85, *SD*_*JPN*_ = 9.99), *t*(31.61) = 1.61, *p* > .10, there was a significant cultural difference in parents’ accounts for the background reference: Japanese parents (*M*_*JPN*_ = 16.40, *SD*_*JPN*_ = 7.43) were more likely than their Canadian counterparts (*M*_*CAN*_ = 5.05, *SD*_*CAN*_ = 5.72) to refer to the background figures’ emotional expressions, *t*(40) = 5.58, *p* < .001; *d* = 1.71. In addition, although Canadian parents and their children’s number of background references was not statistically significant, *t* < 1, *ns*, Japanese parents were more likely than their children to refer to the background figures’ emotional expressions, *t*(38) = 3.07, *p* = .004; *d* = .97 (see Fig. 1).

As we predicted, the results of children’s accounts replicated previous findings by Masuda et al. (2017b) and Senzaki et al. (2016): Children aged 7–8 did not differ in their reasoning styles across cultures by mainly focusing on the center figure’s facial expressions. In contrast, the results of parents’ accounts replicated previous findings with young adult data (Masuda et al. 2008b; Masuda et al. 2012). More importantly, both Japanese and Canadian parents demonstrated culturally dominant modes of attention in their reasoning styles when they engaged the task in front of their children. In other words, they served as a role model for their children to learn the culturally appropriate way of attending to and explaining a scene.

Notably, compared to the significant difference between Japanese children and parents, Canadian children and parents showed similar reasoning styles by mainly describing the target information. We confirmed this pattern by measuring correlations for the foreground and background accounts between children and parents across cultures. Overall, the correlation for the foreground accounts between children and parents was significant, *r*(44) = .54, *p* < .001; the correlation for the background accounts between children and parents was also significant, *r*(44) = .41, *p* = .005. However, these strong correlations mainly came from the similarities between Canadian parents and children’s responses, with a strong correlation for the foreground accounts, *r*(22) = .82, *p* < .001, and a marginally significant correlation for the background accounts, *r*(22) = .39, *p* = .076. The correlations between Japanese parents and children were not significant for either type of account (i.e., the foreground accounts, *r*(20) = .03, *p* = .90, and the background accounts, *r*(20) = .30, *p* = .20). Although we reported the significant correlations between Canadian parents and children, we do not interpret this pattern to be a result of cultural transmission. Rather, we interpret that the result is merely attributable to the fact that there was no major change in the task performance strategy between Canadian parents and children, and the attention to the center figure which is dominant in Canadian parents seems to be a fundamental way of engaging in the task (in fact, Japanese children and Canadian children showed similar patterns in this matter). We admit that it is too early to discuss whether Canadian parents indeed provided advanced cultural messages for their children’s learning. In future research, it is advisable to devise a task that will allow researchers to assess differences in patterns of attention between Canadian children and parents (e.g., devising stimuli by which Canadian children are distracted by peripheral information, but Canadian parents can focus only on the target area in a visual space).

In sum, this study shows no cultural difference in reasoning styles among 7–8 year-old children in Canada and Japan before interacting with their parents, replicating the findings by Masuda et al. (2017b) and Senzaki et al. (2016). This study further demonstrates that parents’ behavior may play a key role in transmitting culturally appropriate response styles to their children by maintaining their culturally shaped responses in front of their children.

## General discussion

The present study attempted to identify whether parents exhibit attentional styles dominant in a given culture in front of their children, even when their children have not fully developed and internalized such attentional styles (the demonstration of which was similar to a study by Fernald and Morikawa 1993). The results confirmed our expectations: while there were no cultural variations in 7–8- year-old children’s modes of attention, parents maintained culturally dominant modes of attention in front of their children when describing facial expressions in a social context.

### Implications

The current findings shed light on the significance of parent–child interactions in children’s cultural learning (Gauvain 2001; Renninger and Granott 2005; Rogoff 1990, 2003; Wood et al. 1976, 1978; Wood and Middleton 1975). Previous research in cultural psychology indirectly suggested that early elementary-school children’s cognitive styles correspond to that of adult members within a culture (e.g., Ishii et al. 2014; Senzaki et al. 2014; Tsai et al. 2007). To date, only a limited number of studies (Senzaki et al. 2016) have examined how interactions between parents and children can facilitate the emergence of culturally dominant attention among children. Further, the present study provided direct evidence of how parents can act as guidance during parent–child interaction by demonstrating how parents’ reasoning style represents a culturally normative pattern of attention.

### Limitations and future research

Although we showed how parents play a role in demonstrating culturally dominant modes of attention during interaction with their children, there remains one question as to whether children would internalize parents’ attentional styles after the observation of parental behavior. Previous studies have compared the extent to which children’s performance improves before and after interacting with their parents in order to pinpoint children’s learning from adult–child interaction (e.g., de la Ossa and Gauvain 2001; Gauvain et al. 2001). Therefore, future research should also employ this paradigm to examine whether cultural variation can emerge after the interaction with parents among children who show no cultural variation before the interaction. Masuda et al. (2017a) have recently applied this comparison design of before and after parent–child interaction by targeting young children from 7 to 9 years old in order to see children’s internalization of parents’ attentional styles.

In addition, although we attempted to capture parental behavior during one joint session, it is worthwhile to examine whether parents adjust their behavior depending on their children’s age. By comparing parental behavior with children in different age groups (e.g., 7–8 vs. 9–10), researchers can detect how parental behavior adapts according to the child’s age. In a previous study, mothers tended to provide different levels of assistance depending on their children’s age: the younger the children were, the more they provided assistance (de la Ossa and Gauvain 2001). This implies the adjustment of parental behavior depending on their children’s stage of development. Thus, future research should address whether the degree to which parents provide assistance to their children becomes less if the children already show the culturally dominant mode of attention.

Although we applied participants’ behavioral data as indicators of their modes of attention, a direct measurement of attention, i.e., eye movements, should be used to strengthen our argument and more accurately capture parents’ and children’s attentional patterns. Researchers have provided supportive evidence of cultural variations in modes of attention by using eye tracking among young adults (e.g., Chua et al. 2005; Masuda et al. 2016; Senzaki et al. 2014). Eye-movement research for children in a cross-cultural context entails a variety of constraints; however, this would capture how attention plays a role during parent–child interaction and can find early development of joint attention patterns that play a pivotal role for children to imitate and internalize adults’ skills and knowledge (e.g., Bruner 1983; Moore and Dunham 1995; Tomasello 1999). By utilizing an eye-tracking method, future research can clarify and illustrate child developmental trajectories of culturally specific modes of attention.
